# Gut microbiota from coronary artery disease patients contributes to vascular dysfunction in mice by regulating bile acid metabolism and immune activation

**DOI:** 10.1186/s12967-020-02539-x

**Published:** 2020-10-09

**Authors:** Honghong Liu, Ran Tian, Hui Wang, Siqin Feng, Hanyu Li, Ying Xiao, Xiaodong Luan, Zhiyu Zhang, Na Shi, Haitao Niu, Shuyang Zhang

**Affiliations:** 1 Department of Cardiology, Peking Union Medical College Hospital, Peking Union Medical College & Chinese Academy of Medical Sciences, 1 Shuaifuyuan, Dongcheng District, Beijing, 100730 China; 2 Institute of Laboratory Animal Sciences, Chinese Academy of Medical Sciences and Comparative Medicine Center, Peking Union Medical Collage, Beijing, 100021 China; 3 School of Medicine, Jinan University, Guangzhou, 510632 China

**Keywords:** Gut microbiota, Faecal microbiota transplantation, Bile acids, Intestinal immunity, Vascular dysfunction

## Abstract

**Background:**

The gut microbiota was shown to play a crucial role in the development of vascular dysfunction, and the bacterial composition differed between healthy controls and coronary artery disease patients. The goal of this study was to investigate how the gut microbiota affects host metabolic homeostasis at the organism scale.

**Methods:**

We colonized germ-free C57BL/6 J mice with faeces from healthy control donors (Con) and coronary artery disease (CAD) patients and fed both groups a high fat diet for 12 weeks. We monitored cholesterol and vascular function in the transplanted mice. We analysed bile acids profiles and gut microbiota composition. Transcriptome sequencing and flow cytometry were performed to evaluate inflammatory and immune response.

**Results:**

CAD mice showed increased reactive oxygen species generation and intensive arterial stiffness. Microbiota profiles in recipient mice clustered according to the microbiota structure of the human donors. *Clostridium symbiosum* and *Eggerthella* colonization from CAD patients modulated the secondary bile acids pool, leading to an increase in lithocholic acid and keto-derivatives. Subsequently, bile acids imbalance in the CAD mice inhibited hepatic bile acids synthesis and resulted in elevated circulatory cholesterol. Moreover, the faecal microbiota from the CAD patients caused a significant induction of abnormal immune responses at both the transcriptome level and through the enhanced secretion of cytokines. In addition, microbes belonging to CAD promoted intestinal inflammation by contributing to lamina propria Th17/Treg imbalance and worsened gut barrier permeability.

**Conclusions:**

In summary, our findings elucidated that the gut microbiota impacts cholesterol homeostasis by modulating bile acids. In addition, the CAD-associated bacterial community was shown to function as an important regulator of systemic inflammation and to influence arterial stiffness.

## Background

Mounting evidence indicates a strong link between intestinal dysbiosis and the development of cardiovascular diseases (CVD). A wide variety of bacterial derivative metabolites have been shown to module vascular pathophysiology, including trimethylamine-N-oxide (TMAO), short-chain fatty acids (SCFAs) and bile acids (BAs) [1, 2]. Cohort studies in diverse populations have demonstrated that bile acids may be a useful biomarker to predict the severity of CVD [3, 4]. Bile acids are synthesized from cholesterol in the liver via the classic pathway initiated by cholesterol 7α-hydroxylase (CYP7A1). The primary bile acids are excreted into intestine and then modified by bacteria through deconjugation, 7α-dehydroxylation and epimerization reactions to produce secondary bile acids, such as lithocholic acid (LCA), hyodeoxycholic acid (HDCA) and ursodeoxycholic acid (UDCA). Approximately 95% of the bile acids are reabsorbed in the ileum and returned to the liver via the enterohepatic cycle, allowing bile acids to function as endocrine-like signalling molecules that modulate host metabolism and energy homeostasis [5]. Although the results of many studies support the notion that gut microbiota drive cardiovascular disease through modifications to bile acids, faecal microbiota transplantation experiments in germ-free mice are still need to explore the chain of causation from various bacterial communities.

The intestinal immune system serves as a gatekeeper to prevent pathogenic invasions and preserve a balanced gut microbiota environment. Microbial dysbiosis can increase gut permeability and enhance the penetration of bacterial-derived endotoxins into circulation, resulting in metabolic endotoxaemia, exacerbated lipid homeostasis and the promotion of vascular remodelling [6, 7]. It is well appreciated that microorganism-associated molecular patterns can promote CVD via the direct engagement of host pattern recognition receptors. Recently, a study showed that gut microbiota and LCA could control host immune responses by directly shaping the balance of Th17 and Treg cells [8]. Overall, gut microbiota can directly engage the immune system not only to elicit the appropriate bactericidal responses but also to regulate inflammatory pathways relevant to CVD.

**Fig. 1:**
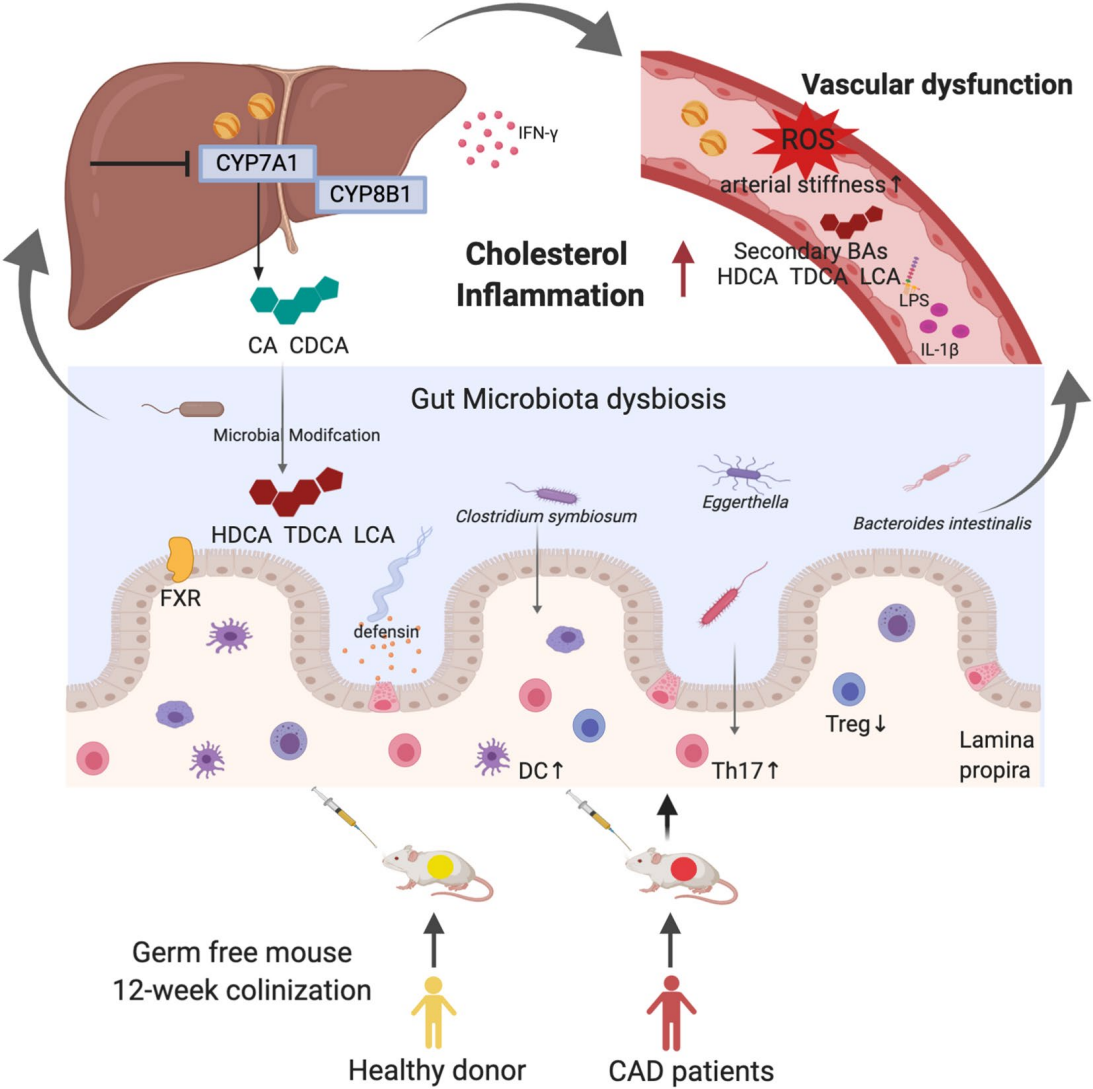
A model depicting the correlation among CAD related microbiota, cholesterol disorder, bile acids profile, abnormal Th17/Treg, and vascular dysfunction. Faecal microbiota from CAD patients induce higher cholesterol level via enriching secondary bile acids synthesis in the recipient germ free mice. Meanwhile, CAD related microbiota stimulate the immune response featured by Th17/Treg imbalance not only in intestinal but also systematically, causing endotoxemia and increased vascular tone. These findings indicated a causal role of microbiota in the pathogenesis of vascular dysfunction

In our previous study, we demonstrated that alterations in the gut microbiota were correlated with CAD severity via changes in taurine levels using multi-omics analyses [9]. In order to understand the exact link between microbiota, bile acids, and prove the causality between gut microbiota and cardiovascular disease, we colonized germ-free mice with faecal microbiota from healthy human donors and CAD patients, to verify whether the intestinal microbiota contributes to a vascular dysfunction phenotype. We demonstrated that the transplanted microbiota played a role in regulating bile acids and cholesterol homeostasis, as well as in facilitating systemic and intestinal immune responses. Finally, the gut microbiota from CAD patients impacted the development of vascular dysfunction after long-term colonization (Fig. 1).

## Materials and methods

### Animals and diets

C57BL/6 J germ-free (GF) mice were generated and provided by the Institute of Laboratory Animal Sciences (ILAS) at the Chinese Academy of Medical Sciences and Peking Union Medical College [research license no. SYXK (Beijing) 2015-0035], which is a member of (and accredited by) the American Association for the Accreditation of Laboratory Animal Care. All experiments were performed in accordance with the guidelines of the Institutional Animal Care and Use Committees of the ILAS. All C57BL/6 J GF mice were female and age matched. All GF mice were used after reaching the age of 8 weeks and were housed independently, with one mouse per cage and one group per isolator. The high fat diet (23% protein, 45% fat, and 20% carbohydrate, 4.5 total kcal/g; product number D12109C) was purchased from Research Diets, Inc. The normal chow diet (24.02% protein, 12.95% fat, and 63.03% carbohydrate, 3.44 total kcal/g) was purchased from Beijing Keao Xieli Feed Co., Ltd.

### Faecal microbiota collection and transplantation

To explore whether the intestinal microbiota has the ability to affect metabolic and cardiovascular function, we colonized germ-free C57BL/6 J GF with faecal microbiota from healthy control donors (Con, N = 5) and coronary artery disease patients (CAD, N = 6) from our previous CAD cohort. Faeces and serum from human donors were separately collected as previously described [9]. The appropriate number of donors was determined on the basis of previous FMT experiments [10, 11]. We conducted donors screening according to the following standards: *i.* We selected CAD patients who were also diagnosed with myocardial infarction, since their clinical symptoms were more severe and typical; *ii*. We screened CAD patients distinguished significantly with healthy donors based on principal co-ordinates analysis (PCoA) using Bray–Curtis distance. This project was approved by the Ethics Committee of the Peking Union Medical College Hospital, Beijing (JS-1195). Written informed consent was obtained from the donors.

Adult GF mice were divided into 2 groups: (*i*) Con group (n = 11), gavaged with faeces from healthy donors and received a high fat diet; and (*ii*) CAD group (n = 12), gavaged with faeces from myocardial infarction patients and received a high fat diet. Additionally, germ-free (GF) mice gavaged with phosphate-buffered saline solution (PBS) that received a normal chow diet were raised as the untreated control group. One gram of each mixed sample was suspended in 5 mL of PBS, vortexed thoroughly, and then administered as a 200-μL aliquot. To ensure the continuous effect of the microbiota transplantation, after 5 oral gavages, once every two days (days 1, 3, 5, 7, and 9), the mice were then orally gavaged once a week until the end of the experiment after 12 weeks.

### Serum and hepatic lipid concentrations

Blood samples were collected from the orbital vascular plexus of mice before they were sacrificed. A frozen pellet of liver sample was homogenized in a corresponding volume (1:9, w/v) of homogenization buffer (0.01 mol/L Tris–HCl, 1 mmol/L EDTA, and 0.8% NaCl, pH 7.4). The supernatant was collected after centrifugation at 2000×*g* for 25 min at 4 °C. Assay kits (NanJing Jiancheng Bioengineering Institute, China) were used to measure the concentrations of hepatic and serum triglyceride and cholesterol, and the hepatic lipid levels were corrected for total protein concentration (BCA protein assay kit, Thermo Fisher Scientific Co., Ltd.).

### Enzyme-linked immunosorbent assay

Blood samples were centrifuged at 3500 rpm and 4 °C for 15 min, and the serum was stored at − 80 °C until further analyses. Tissue pre-treatment methods including ileum and liver were the same as above. Expressions for fibroblast growth factor 15 (Fgf15), LPS, claudin-1 and ZO-1 were measured by enzyme-linked immunosorbent assays (ELISAs) according to the manufacturer’s instructions (Cloud-Clone Corp.). The levels of inflammatory factors in serum were measured using a V-PLEX Pro inflammatory panel following the manufacturer’s instructions.

### UPLC–MS/MS analysis of bile acid profiles

For serum samples, 180 μL of acetonitrile/methanol (8:2) containing 10 internal standards was added to 20 μL of serum sample in a 96-well plate, and metabolite extraction was conducted using a laboratory shaker at 10 °C with shaking at 1500 rpm for 20 min. For each faecal sample, 10 mg faeces was cut and accurately weighed in a SafeLock Eppendorf microcentrifuge tube before being homogenized with approximately 25 mg of pre-chilled beads and 20 μL of ultrapure water using a homogenizer (BB24, Next Advance, Inc., Averill Park, NY, USA). Then, the samples were homogenized with 180 μL of acetonitrile/methanol (8:2) containing 10 internal standards and centrifuged at 13,500 rpm and 4 °C for 20 min.

After centrifugation, the supernatant was transferred to a microcentrifuge tube for lyophilization using a FreeZone freeze dryer equipped with a stopping tray system (Labconco, Kansas City, MO, USA). The dried sample powder and the lyophilized calibrators and QCs from BAP Ultra were reconstituted with of acetonitrile/methanol (80/20, v/v) and ultrapure water (1:1, v/v), and centrifuged at 13,500×*g* and 4 °C for 20 min (Microfuge 20R, Beckman Coulter, Inc., Indianapolis, IN, USA). Then, the supernatant was transferred to a 96-well plate for LC-MS analysis using an injection volume of 5 μL.

An ultra-performance liquid chromatography coupled to tandem mass spectrometry (UPLC-MS/MS) system (ACQUITY UPLC-Xevo TQ-S, Waters Corp., Milford, MA, USA) was used to quantify bile acid levels in this study. The optimized instrument settings and analytical quality control procedures are briefly described in Additional file 2.

### Analysis of metagenomics data

#### Microbial DNA extraction and metagenomic sequencing

The mouse faecal samples were collected the day before sacrifice. DNA extraction was performed using a Qiagen QIAamp DNA Stool Mini kit (Qiagen) following the manufacturer’s instructions. Extracts were treated with DNase-free RNase to remove contaminating RNA, and DNA quantity was determined using a NanoDrop spectrophotometer, a Qubit Fluorometer (with the Quant-iTTMdsDNA BR Assay kit), and gel electrophoresis. DNA library construction was performed following the manufacturer’s instructions (Illumina). The average fragment size in the final DNA libraries was determined using an Agilent 2100 Bioanalyzer (Agilent Technologies, USA) and quantified using an ABI StepOnePlus Real-Time PCR system (Applied Biosystems, USA). All samples were sequenced using an Illumina HiSeq X Ten platform with an insert size of 300 bp (paired end, 150 bases pairs). After removing adaptors and low-quality reads, the remaining reads were filtered to eliminate the host DNA genome.

#### De novo non-redundant metagenomic gene-catalogue construction

High-quality paired-end reads from each sample were used for de novo assembly with IDBA_UD v1.1.0 [12] into contigs longer than 300 bp. The assembly results were detailed in Additional file 1: Table S4. The high quality reads from each sample were aligned against the gene catalogue by SOAP2 v2.22 [13] using the criterion of “identity > 90%”. The prediction of genes from each sample was performed using Meta-GeneMark v.2.1 [14]. A non-redundant gene catalogue of 440,930 microbial genes was constructed with CD-HIT v4.6.4 using the parameters “-c 0.95 -aS 0.9”. The high-quality read distribution of different samples was aligned with Bowtie2 with the parameters “-p8 –very-sensitive-local -k 100 –score-min L,0,1.2” [15]. The gene abundance was used to calculate alpha and beta diversity. KEGG orthology (KO) assignments were made using the same procedure as described previously [16].

#### Functional and taxonomic annotation

Putative sequences were translated from the gene catalogue and aligned against the proteins/domains in the KEGG databases (release 89.1, with animal and plant genes removed) using BLASTp (v2.2.24, default parameter except that -e1e -5a6 -b50 -FFm8). Each protein was assigned to a KO by the highest scoring annotated hit(s) containing at least one high-scoring segment pair (HSP) scoring over 60 bits. MEGAN v4.6 [17] was used taxonomic annotation and species abundance statistics. We used the ReporterScore [18] method to statistically analyse all related KOs and used the overall trend to reflect the change in the pathway. Genes and KEGG orthology genes are linked in KEGG. For each of the KO genes, the abundance of microbial genes was summed so that each component in each KO gene represents an organism.

#### Diversity analysis

α-diversity (within-sample diversity) was calculated using the Shannon index depending on the gene and taxonomy profile. β-diversity (between-sample diversity) was estimated by Bray–Curtis dissimilarity. The significance of differences in gut microbiota among different groups was assessed by permutational multivariate analysis of variance (PerMANOVA test with 999 permutations) in R (3.6.0). Spearman correlations were calculated using an R package, and all differential abundances of genes, species and KO screening were tested using the Wilcoxon rank sum test. Where indicated, the Benjamini–Hochberg method was used to control the FDR.

#### Isolation of total RNA and quantitative reverse transcription PCR (RT-qPCR) analysis

Total RNA was extracted from the ileum and liver using an RNeasy^®^ Mini kit following the manufacturer’s instructions. cDNA synthesis was performed using PrimeScript™ RT Master Mix (TaKaRa Bio., Japan). Then, qRT-PCR was performed using an Applied Biosystem StepOne Plus PCR system with TB Green™ *Premix Ex* Taq™ II (TaKaRa Bio., Japan). Gene expression levels were determined using the comparative ΔΔC_T_ method, and the sequences of the primers used in this study are shown in Additional file 1: Table S10.

#### Transcriptome sequencing

Total RNA in mouse livers was extracted using RNeasy mini kit (Qiagen, Germany). The concentration and quality of the purified RNA was determined using a NanoDrop ND-1000 instrument (Thermo Scientific, United States) and an Agilent Bioanalyzer 2100 system (Agilent Technologies, United States), respectively. The sequencing libraries were generated according to the Illumina TrueSeq protocol. Paired-end RNA sequencing was performed on an Illummina HiSeq 2500 instrument following the manufacturer’s instructions with a quality control standard (for each sample, the sequencing depth was higher than 6 GB, the read length was higher than 90 nt, and the Q30 > 85%). Raw data (raw reads) in the fastq format were first processed using in-house Perl scripts. Clean data (clean reads) were then obtained by removing reads containing adapter sequences and poly-N sequences from the raw data.

The high-quality reads from each sample were aligned to the Mus musculus reference genome, GRCm38 (Genome Reference Consortium mouse build 38), using hierarchical indexing for spliced alignment of transcripts (HISAT2) with the default parameters [19]. The unambiguous alignments from each sample were quantified using *featureCounts* [20], and the gene expression profile with respect to the read counts was acquired. Differentially expressed genes in the two groups were determined by using the *DESeq* *2* package [21] in R. Gene ontology (GO) enrichment and (Kyoto encyclopedia of genes and genomes (KEGG) pathway analysis of differentially expressed genes was performed using the *clusterProfiler* package [22]. Significant differences are presented as the adjusted *P* value.

#### Isolation of splenocytes and lamina propria lymphocytes (LPLs)

Spleens were removed and cut into pieces in cold PBS. After filtration through a 200-gauge steel mesh and the removal of red blood cells with RBC lysis buffer (BD Biosciences, USA), splenocytes were collected for further experimentation. The isolation of small intestine LPLs was performed as previously described [23].

#### Flow cytometry

Splenocytes and siLPLs were incubated with a rat anti-mouse CD16/CD32 mAb (BioLegend, USA) for 30 min to block Fc receptors. Then, the cells were stained on ice with fluorochrome-conjugated monoclonal antibodies for 30 min as previously described [24]. To perform intracellular staining, cells were treated using a Fixation/Permeabilization Solution kit (BD Pharmingen™, USA) and then stained with the appropriate antibodies. The antibodies used in this study are listed in Additional file 2: Figure S6. Cells were assayed with a Symphony A5 cytometer (BD Biosciences), and the resulting data was analysed using FlowJo (ver. 10.4).

#### Pulse wave velocity measurement

The pulse wave velocity (PWV) in the left common carotid artery of mice was noninvasively measured using a regional transit-time (TT) method as previously described [25]. Transit-time measurements were performed using a Visual Sonics Vevo770 ultrasound biomicroscope (Visual Sonics Inc., Toronto, ON, Canada), and the results were processed using VISUAL SONICS analysis software.

### Histological analysis and reactive oxygen species detection

For immunohistochemical staining analyses, the aorta (from the arch to the thoracic aorta) of each mouse was serially sectioned (6-μm sections, 40 sections per mouse). The collagen content in the tissue was assessed by Masson’s trichrome staining, and reactive oxygen species (ROS) generation was measured by the detection of fluorescent dihidroethidium (DHE) oxidation products. In the presence of superoxide and other reactive species, DHE is oxidized to 2-hydroxyethidium and ethidium, which are trapped by intercalation with DNA, producing bright red fluorescence [26]. The tissues were scanned with Pannoramic DESK and analysed using Image Pro Plus 6.0. Quantitative estimations of histochemical staining were carried out independently by two blind investigators. For quantification by image analysis, we set a threshold to automatically compute the areas positive for collagen (blue signal for Masson’s trichrome) or ROS signal and then computed the percentage of positively stained area to the total cross-sectional vessel wall area. Fluorescence fields were evaluated in at least six different locations in each image and results averaged per mouse.

### Statistical analysis

Statistical analysis was performed using Prism version 7 (GraphPad Software, Inc.) and SPSS 20.0. The data are presented as medians (interquartile range) or mean ± SD. Statistical comparisons were performed using Mann–Whitney, unpaired T test and Kruskal–Wallis tests as appropriate. The Benjamini and Hochberg false discovery rate correction method was used when multiple comparisons were performed. *P* < 0.05 was considered significant.

## Results

### Faecal microbiota from CAD patients influence lipid homeostasis in GF mice

**Fig. 2:**
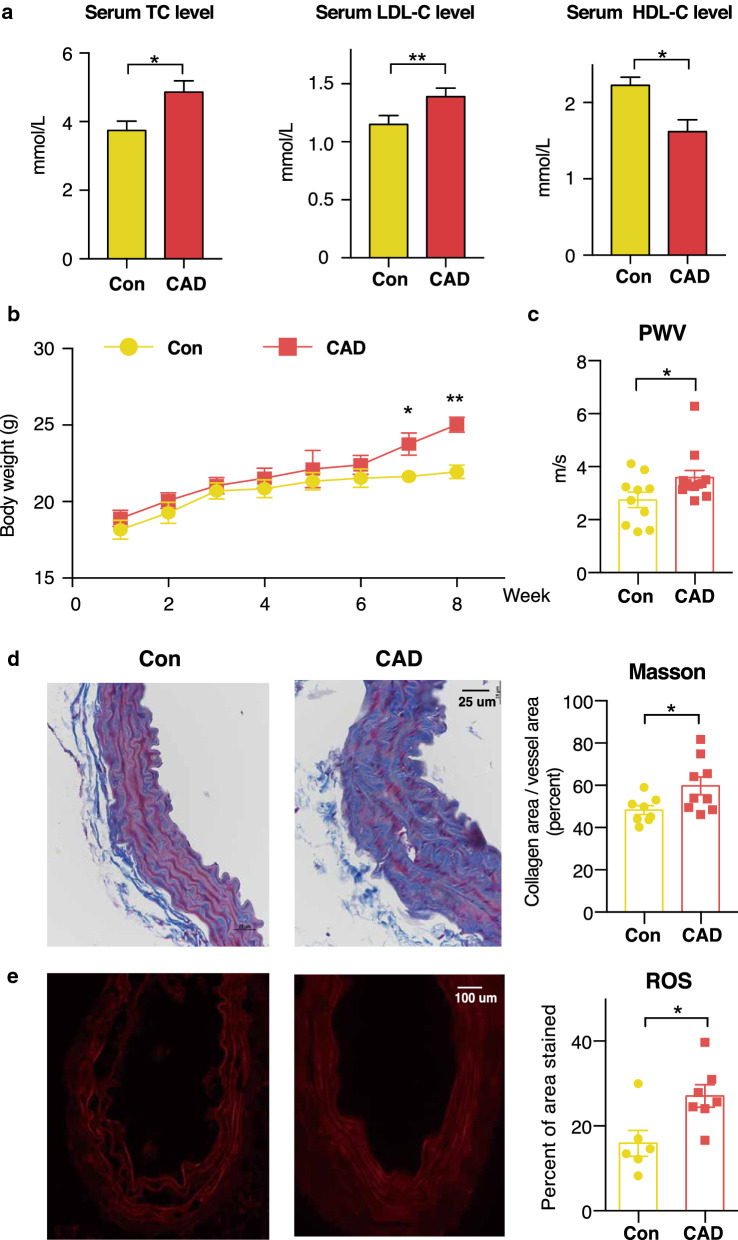
Effect of colonization with microbiota from CAD patients and healthy control donors on cholesterol imbalance and vascular stiffness in germ-free mice. **a** Serum TC, LDL-C and HDL-C level. **b** Body weight curve of Con and CAD groups during 8-week. **c** CAD mice show increased arterial stiffness. The data was collected from pulse wave velocity assay in left common carotid artery and analyzed using commercial software. **d** Mouse aorta sections were collected. Masson’s trichrome staining was performed to determine the positive area of collagen. **e** Analysis of dihydroethidium (DHE) derived fluorescence and quantification show enhanced ROS generation in aorta of CAD mice (n = 7) compared to Con mice (n = 6). Mean ± SEM are plotted, *P < 0.05, **P < 0.01, Mann–Whitney U test. Con mice, n = 8-11; CAD mice, n = 9-12. TC, total cholesterol; LDL-C, low density lipoprotein-cholesterol; HDL-C, high density lipoprotein-cholesterol; PWV, pulse wave velocity; ROS, reactive oxygen species; CAD, colonization with microbiota from coronary artery disease patients;* Con*, colonization with microbiota from healthy donors

To evaluate how long-term gut microbiome transplantation impact cholesterol homeostasis, 6-week-old germ-free C57BL/6 J mice were randomly assigned to one of the following three groups: (*i*) Control group (Con), gavaged with faeces collected from healthy donors and received a high fat diet; (*ii*) Coronary artery disease group (CAD), gavaged with faeces collected from CAD patients and received a high fat diet (Fig. 1). Additionally, germ-free (GF) mice gavaged with PBS and received a normal chow diet were raised as non-treatment control group. We observed that individuals from CAD group were characterized as having a larger waistline and increased fasting blood glucose and inflammatory factor levels, including hs-CRP, IL-1β and TNF-α (Table 1). However, the CAD patients did not show an elevated blood lipid profile due to statin usage. After long term faecal microbiota transplantation (FMT) with germ-free mice, the serum levels of total cholesterol (TC) and low-density lipoprotein-cholesterol (LDL-C) in CAD mice were significantly elevated compared to those observed in the Con mice, whereas Con mice showed higher levels of high-density lipoprotein cholesterol (HDL-C) (Fig. 2a). We observed gut microbiota modulated hepatic HDL-C levels in CAD mice which may be caused by the dietary lipid composition [27] (Additional file 1: Figure S1). And the CAD mice exhibited an increase in body weight during the 12-week (Fig. 2b). Overall, these results indicated that the microbiota from CAD patients could regulate cholesterol homeostasis in C57BL/6 J germ-free mice.

**Table 1:** The clinical information of faecal microbiota transplantation donors

	Con donor (n = 5)	CAD donor (n = 6)	P value
Gender (Male)^c^	3 (60%)	4 (66.67%)	0.819
Age^b^	53.8 ± 8.1	66 ± 14.23	0.178
BMI^b^	22.3 ± 4.2	25.7 ± 5.1	0.247
Waist^b^	76.4 ± 10.4	93.5 ± 12.1	0.035
Gensini score^a^	NA	32 (26.75, 46.25)	
Current smoker^c^	2 (40%)	4 (66.67%)	0.377
Hypertension^c^	1 (20%)	3 (50%)	0.122
Type 2 diabetes^c^	0	2 (33.33%)	0.154
TC^b^	4.6 ± 0.5	4.4 ± 1.6	0.74
TG^b^	1.1 ± 0.2	2.1 ± 0.8	0.338
LDL-C^b^	2.8 ± 0.2	2.6 ± 0.4	0.662
HDL-C^b^	1.27 ± 0.2	0.9 ± 0.1	0.092
FBG^b^	1.27 ± 0.2	10.23 ± 2.5	0.005
hs-CRP^b^	5.82 ± 0.6	38.5 ± 17.1	0.008
IL-1β^b^	2.66 ± 0.3	3.44 ± 0.9	0.030
TNF-α^b^	8.11 ± 5.4	20.4 ± 6.1	0.007
Drugs			
ACEI/ARB^c^	1 (20%)	3 (50%)	0.303
Antidiabetic drugs^c^	0	2 (33.33%)	0.154
Statin^c^	0	4 (66.7%)	0.022

^a^Median (IQR), ^b^mean ± SD, ^c^n (%)

Continuous, normally distributed variables between two groups were analysed by Student’s t-test. Categorical variables were compared by the χ^2^ test. *N/A* not available, *Gensini Score* scoring system for determining the severity of coronary heart disease, *BMI* body mass index, *TC* total cholesterol, *TG* triglyceride, *LDL-C* low density lipoprotein cholesterol, *HDL-C* high density lipoprotein cholesterol, *FBP* fasting blood glucose, *hs-CRP* high sensitive C-reactive protein, *IL-1β* interleukin 1β, *TNF-α* tumor necrosis factor-α, *ACEI/ARB* angiotensin-converting enzyme inhibitors/angiotensin receptor blockers

### Faecal microbiota from CAD patients cause arterial stiffness and vascular dysfunction

The gut microbiome has been shown to play an important role in oxidative stress and inflammation, which can influence vascular disease [28]. We sought to study the impact of CAD microbiota on vascular tone in unchallenged animals in a C57BL/6 J background. We used pulse wave velocity (PWV) measurements to assess aortic stiffness, and the mice transplanted with the CAD microbiota showed enhanced vascular stiffness (Con: 2.75 ± 0.29 m/s vs. CAD: 3.59 ± 0.27 m/s; *P* = 0.043, Fig. 2c, Additional file 2: Figure 2). Collagen deposition as a contributor to vascular remodelling was also investigated. We observed elevated levels of collagen deposition in the aortas of CAD mice (*P *= 0.043, Fig. 2d). As the generation of reactive oxygen species (ROS) can trigger the apoptosis of endothelial cells and alter vasomotor functions [29], we determined the amount of vascular superoxide anions using immunofluorescence, and the CAD mice showed globally increased superoxide generation in the aorta (*P* < 0.001, Fig. 2e). Taken together, these results indicated the CAD-associated microbiota promoted vascular structural changes caused by intravascular ROS and vascular fibrosis.

### GF mice colonized with microbiota from CAD patients exhibit imbalanced bile acid profiles

**Fig. 3:**
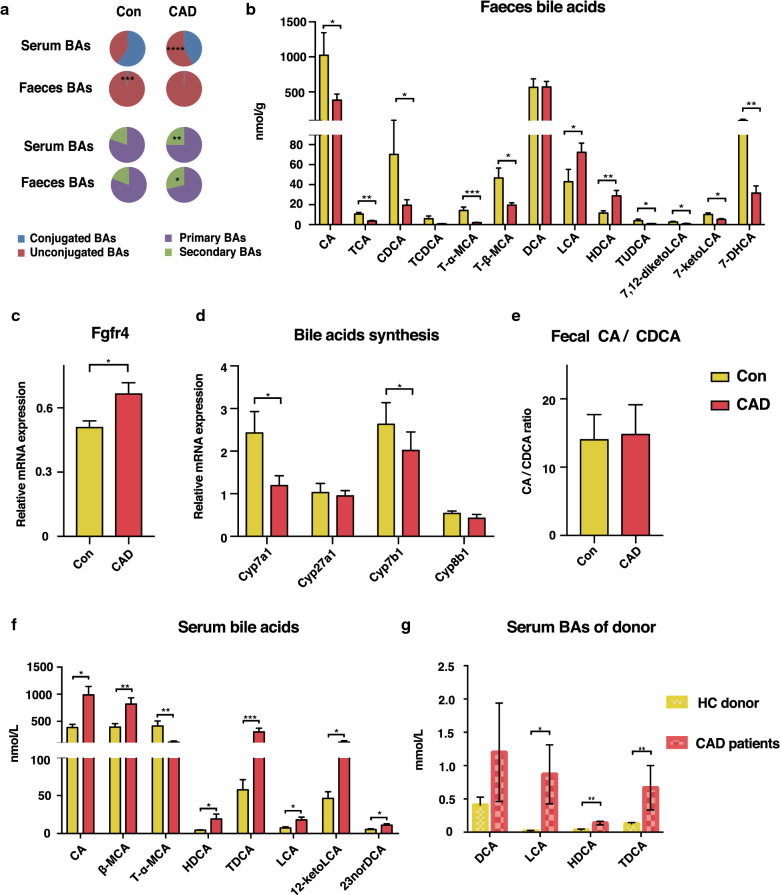
Microbiota from CAD patients cause imbalanced bile acids profiles and regulate genes involved in BA synthesis. **a** Relative amounts of bile acids categories in the serum and faeces of Con and CAD mice. **b** UPLC-MS/MS analyses of bile acids in the faeces significantly changed. **c** Gene expression of Fgfr 4 in the liver. **d** The graphs represent gene expression of enzymes involved in bile acids biosynthesis in the liver. **e** The proportion of CA/CDCA ratio in the faeces. **f** Concentrations of bile acids significantly changed in the serum. **g** Secondary bile acids abundance in CAD patients (N = 6) and healthy volunteer donors (N = 5). Mean values ± SEM are plotted; *P < 0.05, **P < 0.01, ***P < 0.001, ****P < 0.0001, Mann–Whitney U test. *Con* colonization with microbiota from healthy donors, n = 9-11; CAD, colonization with microbiota from coronary artery disease patients, n = 10-12. BAs, bile acids; CA, cholic acid; CDCA, chenodeoxycholic acid; MCA, muricholic acid; DCA, deoxycholic acid; HDCA, hyodeoxycholic acid; LCA, lithocholic acid; UDCA, ursodeoxycholic acid; T, taurine-conjugated

To assess the regulation of lipid metabolism pathways by the gut microbiota, we focused on bile acids, which are synthesized from cholesterol and converted to secondary bile acids by gut microbiota. To this end, we quantified more than 20 different bile acids in the faeces and serum (Additional file 1: Tables S1 and S2). In serum, the proportions of unconjugated bile acids (BAs) and secondary BAs were higher in the CAD mice than those observed in the Con mice. Similarly, the CAD group also exhibited a higher proportion of secondary bile acids in the faecal bile acid pool, indicating that the microbial metabolism of BAs in CAD mice lead to increased BA diversity (Fig. 3a). The faeces from Con mice had higher levels of cholic acid (CA) and chenodeoxycholic acid (CDCA) than those observed in the CAD mice, indicating that the gut microbiota from healthy donors deconjugated taurine-conjugated bile acids in the small intestine more effectively. We observed that gut microbiota variation had little effect on basolateral transporter (Ostα), apical bile acid transporter (Ibat), and ileal bile acid-binding protein (Ibabp) expression, although Ibat expression was upregulated in the GF mice (Additional file 2: Figure S3a). The primary bile acids that escape reabsorption enter the colon and are further metabolized by microbiota and transformed into secondary BAs. We observed that Con mice had greater abundances of tauroursodeoxycholic acid (TUDCA), 7,12-diketolithocholic acid (7,12-diketoLCA), 7-ketolithocholic acid (7-ketoLCA) and 7-ketodeoxycholic acid (7-DHCA) than was detected in CAD mice, while the microbiota in the CAD mice induced the production of higher levels of hyodeoxycholic acid (HDCA) and lithocholic acid (LCA) (Fig. 3b).

Farnesoid X receptor (Fxr) has emerged as a key regulator in the control of bile acid metabolic homeostasis. TαMCA and TβMCA have also been identified as naturally occurring Fxr antagonists, whereas CA and CDCA are the most effective stimulants for Fxr [30]. We observed that Fxr expression in the ileum of Con mice was higher than that detected in the CAD mice; this outcome may be due to the regulation of multiple bile acids. The expression of fibroblast growth factor 15 (Fgf15), a molecular target of Fxr, was also increased in the Con group (Additional file 2: Figure S3b and c). Fgf15 produced in the distal small intestine was previously shown to bind fibroblast growth factor receptor 4 (Fgfr4) in hepatocytes and inhibit the expression of the Cyp7a1 gene. Surprisingly, we observed that expression of Fgfr4 was significantly upregulated in the liver of CAD mice (Fig. 3c). The gut microbiota regulates the expression of hepatic enzymes involved in bile acids synthesis, including Cyp7a1, Cyp27a1 and Cyp7b1, where Cyp7a1 is a rate-limiting enzyme for bile acid synthesis [31]. We confirmed that the Con mice had the highest level of total bile acids among the assayed groups, this outcome was consistent with the observed trend of Cyp7a1 expression level in the liver (Fig. 3d). In particular, the ratio between the primary bile acids CA and CDCA (CA/CDCA ratio) is determined by Cyp8b1 [32]. We observed no difference in the CA/CDCA ratio between Con and CAD groups nor the mRNA level of Cyp8b1 (Fig. 3d and e), consistent with a previous report that Cyp8b1 is not under microbial regulation [33].

We also analysed the bile acids composition in the serum, which contains reabsorbed BAs from the intestine. We observed the levels of bile acids such as CA, β-MCA, HDCA, taurodeoxycholic acid (TDCA), LCA, 12-ketolithocholic acid (12-ketoLCA) and 23-nordeoxycholic acid (23norDCA) were significantly increased in the CAD mice, especially compared to the Con mice 12 weeks after colonization with the microbiota (Fig. 3f). More importantly, CAD patients also showed higher serum levels of LCA, HCA and TDCA than the HC donors (*P* = 0.017, *P* = 0.002, and *P* = 0.004, respectively, Fig. 3g). Taken together, the gut microbiota from CAD could regulate the composition of the secondary bile acids pool and caused elevated circulatory levels of LCA, HDCA and TDCA. In addition, colonization with the CAD gut microbiota also inhibited the feedback loop of bile acids synthesis, although no activation of the Fgf15-Fgfr4 signalling pathway was observed.

### Faecal microbiota from CAD patients possess enhanced secondary bile acid biotransformation potential

**Fig. 4:**
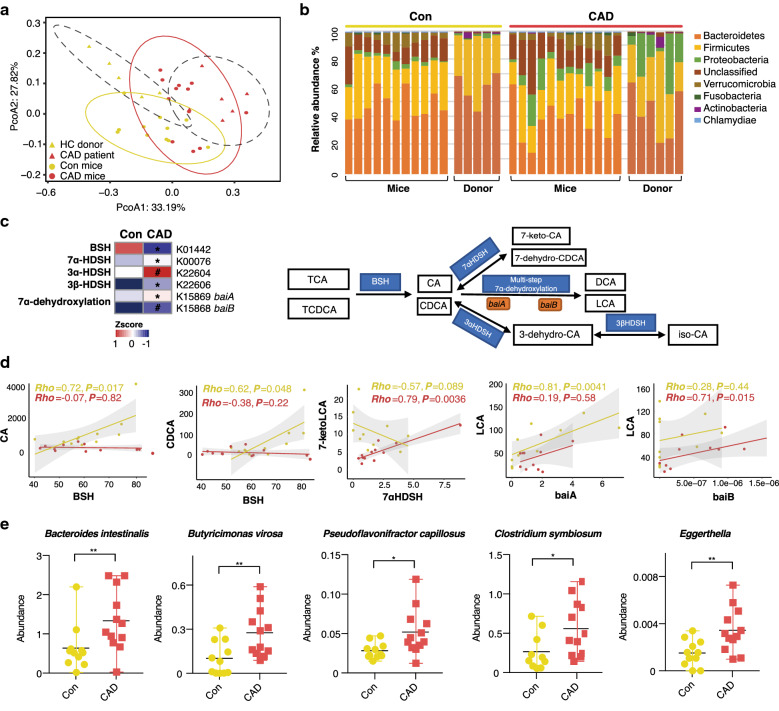
Gut microbiota variation and association with BAs metabolism after faecal microbiota transplantation. **a** Barterial communities from human donors and recipient mouse were clustered using PCoA analysis of the Bray–Curtis distance matrix. **b** Phylum-level changes of human donors and recipient mouse, showing average percentages of each phylum as a proportion of the whole community. **c** Relative abundance of KO genes involved in secondary bile acids metabolism that showed significant difference between Con and CAD groups are shown in the heat map. Significant changes (elevation and depletion) are denoted as follows: *P < 0.05; #*P* < 0.01. Representative KO genes are shown in pathway modules modified from KEGG pathway maps ‘Secondary bile acid metabolism’. **d** Spearman correlations between bacterial relative gene abundance and faecal bile acid levels. **e** Abundance of species between Con versus CAD comparison contributing to secondary BAs transformation. Boxes represent the inter-quartile ranges, and lines inside the boxes denote medians. *P < 0.05, **P < 0.01, Wilcoxon rank sum test. Con mice, n = 10-11; CAD mice, n = 11-12. BSH, bile salt hydrolase; HDSH, hydroxysteroid dehydrogenases; BAs, bile acids; CA, cholic acid; CDCA, chenodeoxycholic acid; DCA, deoxycholic acid; LCA, lithocholic acid; Con, colonization with microbiota from healthy donors; CAD, colonization with microbiota from coronary artery disease patients

We performed metagenomic shotgun sequencing to investigated the transplanted gut microbiota variation. After removing low-quality reads and human DNA reads, on average, 85.2 million high-quality sequencing reads were obtained per sample, which allowed, on average, 98.1% of the reads in each sample to be mapped (Additional file 1: Table S3). We assessed the global structure of the gut microbiota between the mice with different microbiota transplantations. The Con group showed a notable reduction in gene richness (the number of genes identified per mice) compared to that observed in the CAD group (*P* = 0.011, Wilcoxon rank sum test, Additional file 2: Figure S4a). The Shannon index value at the genus level was also lower in the Con group, which was inconsistent with the general belief that higher diversity represents better health (*P* = 0.035, Wilcoxon rank sum test, Additional file 2: Figure S4b) [34]. The overall structure of the microbial community was constructed by PCoA (principal coordinate analysis) based on Bray–Curtis distances. Although no significant taxonomic trend distinguishing the microbiota of CAD patients from that of the healthy controls was observed, in either human donors or the transplanted GF mouse recipients, the microbiota profiles for the mouse recipients were shifted with respect to their human donors (Fig. 4a). However, we observed that taxonomic variations in the recipient mice were consistent among the samples from the donor. At the phylum level, the majority of the bacteria were observed to belong to Bacteroidetes (56.5% Con vs. 35.8% CAD, *P* = 0.045), Firmicutes (37.4% Con vs. 22.5% CAD, *P* = 0.04) and Proteobacteria (2.5% Con vs. 5.1% CAD, *P* = 0.01; Fig. 4b).

We aligned a catalogue of 440,930 nonredundant genes to the Kyoto Encyclopedia of Genes and Genomes (KEGG) database to investigate microbial functions, primarily focusing on bile acids metabolism. Overall, microbial pathways, including amino acids (alanine, aspartate and glutamate) metabolism, biotin and vitamin B6 metabolism were enriched in the Con mice, whereas inflammation-related pathways, consisting of bacterial chemotaxis, biofilm formation, and bacterial secretion system were enriched in the CAD group (Additional file 1: Table S5, Additional file 2: Figure S4c). These functional patterns were similar with those observed in a previous cardiovascular disease cohort study [35]. Next, we examined KO (KEGG orthology) gene abundances to assess the roles of the microorganisms in secondary BA metabolism and manually constructed the pathway in our study by modifying KEGG map00121 (Fig. 4c). Bile acid deconjugation in the gut is performed by bacteria with bile salt hydrolase (Bsh) activity [36]. We determined that the *bsh* gene abundance was elevated in Con mice and was positively correlated with increased faecal CA (*Rho* = 0.72, *P* = 0.017) and CDCA (*Rho* = 0.62, *P* = 0.048) contents. The deconjugated CA/CDCA may subsequently enter the colon, where they are metabolized by microbiota through a multi-step 7-dehydroxylation process into DCA/LCA [37]. The expression of bile acid-inducible genes (*baiA* and *baiB)* involved in the 7-dehydroxylation process was increased in the CAD mice and were observed to be positively correlated with faecal LCA levels. Furthermore, *baiB* showed a stronger association with LCA in the CAD mice (*Rho* = 0.71, *P* = 0.01). Similarly, the microbiota analysis of donors also demonstrated that bacteria community in CAD patients harboured abundant *baiB* gene and was positively associated with serum LCA level in a more significant way (Additional file 2: Figure S4d). In addition, the expression of microbial hydroxysteroid dehydrogenases (HSDHs), which catalyse keto-LCA biotransformation, was significantly elevated in the CAD mice, and the expression of *7αHDSH* in CAD mice was strongly associated with 7-ketoLCA abundance (Fig. 4c and d).

We then examined taxa with the ability of producing secondary BAs. The gene encoding 7α-HDSH was prominently detected in multiple species, including *Bacteroides intestinalis*, *Butyricimonas virosa* and *Pseudoflavonifractor capillosus*, the abundances of which were increased in the CAD mice. In addition, *Clostridium symbiosum* and *Eggerthella*, which contribute the most to 7α-dehydroxylation activity, were more abundant in the CAD mice (Fig. 4e). Clostridium was previously shown to play an important role in producing secondary bile acids [38]. Moreover, *Clostridium symbiosum*, *Clostridium sp. ASF502* and *Butyricimonas virosa* exhibited remarkable positive associations with serum cholesterol levels (Additional file 1: Table S6, Additional file 2: Figure S4e). In summary, the microbiota profiles of the recipient mice were similar to the corresponding donor, and the abundance of intestinal bacteria catalysing LCA and 7-ketoLCA production were significantly increased in the CAD group.

### Faecal microbiota from CAD patients promote systematic and intestinal immune activation of a Th17 phenotype

**Fig. 5:**
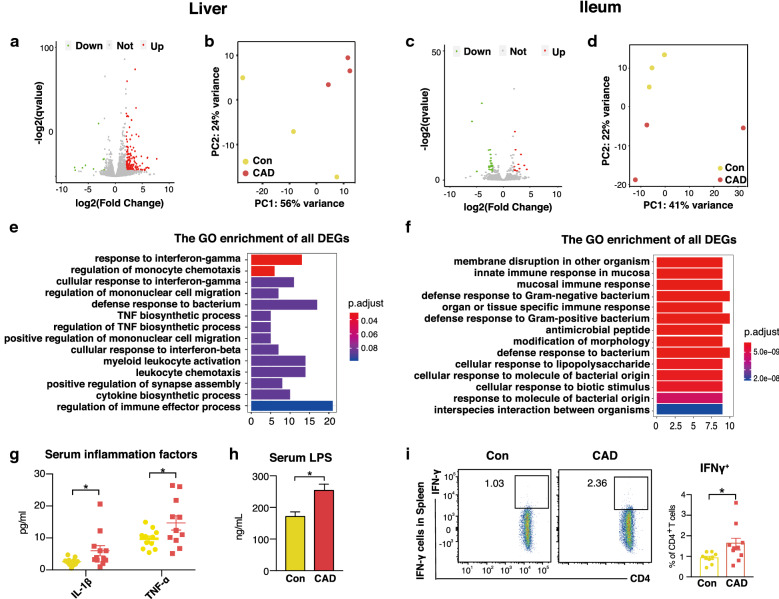
Faecal microbiota from CAD patients induce systematic immune activation and IFN-γ response. **a**, **c** Volcano plot showing pairwise comparisons of differential expression of genes (DEGs) between Con (n = 3) versus CAD (n = 3) mice in the liver and ileum, respectively. Dots represent genes that are significantly different (|Log2(foldchange)| > 2, adjust *P* value < 0.05). **b**, **d** PCA scatter plot of DEGs in the liver and ileum between Con (n = 3) and CAD (n = 3) mice, respectively. **e**, **f)** Results of gene ortholog (GO) enrichment analysis in the liver and ileum, respectively. Bar indicate gene counts for each GO term. **g** Serum inflammatory factors including IL-1β and TNF-α in Con and CAD group. n = 11 or 12 per group. **h** Serum LPS level. n = 11 or 12 per group. **i** CD4^+^IFN-γ^+^ T cells in the spleen (left) and percentages of CD4^+^IFN-γ^+^ T cells within CD4^+^ T cells (right). *P < 0.05, **P < 0.01, Mann–Whitney U test. Con mice, n = 9, CAD mice, n = 10

To assess the biological processes and mechanisms mediated by microbiota from CAD patients, the hepatic and ileal transcriptional response was monitored by transcriptional sequencing. Two hundred twenty-seven differentially expressed genes (DEGs) were identified to be significant in the hepatic transcriptional profile, while only 37 DEGs were identified in the ileal transcriptional profile (Fig. 5a, c, Additional file 1: Tables S7 and S8). PCA analysis was performed with the normalized counts, and the results indicated a clear difference between the transcriptome profiles of the two groups. (Fig. 5b, d). To gain further insight into the metabolic processes differing between the two conditions, functional enrichment analysis was performed (based on biological process GO terms and KEGG pathways). In the liver, biological processes related to inflammatory reactions such as “response to IFN-γ”, “TNF biosynthetic process” and “cytokine biosynthetic process” were significantly elevated in the CAD mice (Fig. 5e and Additional file 2: Figure S5a). In contrast, in the ileum, the most prominent biological processes were immune patterns such as “mucosal immune response”, “antibacterial response” and “cellular response to lipopolysaccharide” (Fig. 5f, Additional file 2: Figure S5b). Subsequently, the levels of 10 cytokines and chemokines were measured in serum samples using a multiplex assay. IL-1β and TNF-α were highly expressed in the CAD mice, and this high expression was consistent with that observed in the human donors (Fig. 5g, Additional file 1: Table S9); in addition, the serum LPS levels were elevated in the CAD group (Fig. 5h).

**Fig. 6:**
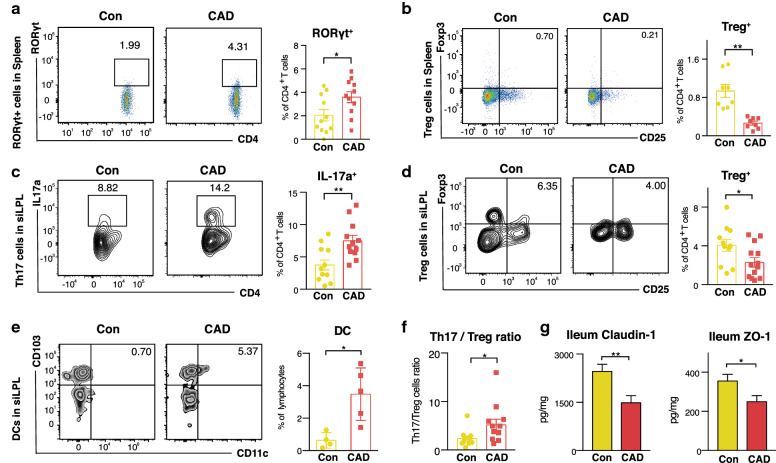
CAD associated microbiota evoke Th17 response and impaired gut permeability in the small intestine. **a** Proportions of RORγt^+^ T cells within the CD4^+^ T cells compartment in the spleen. n = 11 per group. **b** Plots of Tregs gated on CD4^+^CD8a^−^CD25^+^Foxp3^+^ (left); the percentages of Tregs in spleen (right). n = 8 per group. **c** Proportions of Th17 cells (CD4^+^CD8a^−^IL17a^+^) within the CD4^+^ T cells compartment in the siLPL. n = 11 or 12 per group. **d** Plots and percentages of Tregs in the siLPL. n = 11 or 12 per group. **e** Plots and percentages of DCs (CD11c^+^CD103^+^) in the siLPL. n = 4 or 5 per group. **f** Th17/Treg cell count ratio of siLPLs. n = 10 or 11 per group. **g** Claudin-1 and ZO-1 expressions in the ileum. n = 11 or 12 per group. Mann–Whitney t-test was used to compare groups, and error bars represent Means ± SEMs. *P < 0.05, **P < 0.01. siLPL, small intestinal lamina propria lymphocytes; DC, dendritic cells; Tregs, regulatory T cells

Subsequently, we performed flow cytometric analysis on spleen and small intestine samples to explore how the immunological imbalance was affected by microbiota from CAD patients. The complete gating strategy used in this assay is shown in (Additional file 2: Figure S6). Initial characterization of both the spleen and small intestinal lamina propria lymphocytes (siLPLs) populations showed that CD4^+^ T-lymphocytes were increased in the CAD mice (Additional file 2: Figure S7). As we showed that the IFN-γ response was increased in the CAD group, which is a crucial step in immunological defence against bacteria, we assessed the abundance of CD4^+^IFN-γ^+^ T cells (CD3e^+^CD4^+^ CD8a^−^ IFN-γ^+^) and observed that they were increased in the spleens of CAD mice (Fig. 5i), whereas no significant differences were in the distribution of siLPL (Additional file 2: Figure S8a). Then, we investigated the effects of the CAD microbiota on Th17 cells and regulatory T cells (Tregs), which are important players in determining immune balance, mucosal barrier integrity and host protective functions [39]. Flow cytometric analysis of splenocytes revealed that although the proportion of Th17 cells (CD3e^+^CD4^+^CD8a^−^IL17a^+^) was not significantly increased, mice in the CAD group exhibited a distinct shift towards T-cells expressing RORγt (CD3e^+^CD4^+^CD8a^−^RORγt^+^), a master transcription factor that can direct the differentiation of Th17 cells (Fig. 6a) [40]. In contrast, the different microbiota transplantations also altered the distribution of Tregs (CD3e^+^CD4^+^CD8a^−^CD25^+^Foxp3^+^), which were less abundant in the CAD group than in the Con group (Fig. 6b).

Next, we examined the lymphocyte distribution in the lamina propria of the small intestine between the Con and CAD mice. We observed that both Th17 cells (CD3e^+^CD4^+^CD8a^−^IL17a^+^) and CD4^+^RORγt^+^T cells (CD3e^+^CD4^+^CD8a^−^RORγt^+^) were more abundant in the CAD mice (Fig. 6c and Additional file 2: Figure S8b), and CAD mice were characterized as having a significant reduction in Treg cells at the siLPL site (Fig. 6d). Dendritic cells (DCs, CD103^+^CD11c^+^) located at the lamina propria site were present a higher frequency in the CAD mice (Fig. 6e), and these cells have been shown to induce the development of Treg cells, thereby mediating tolerance  [41]. Moreover, the Th17/Treg cell ratio was higher in the siLPL of CAD mice, indicating the occurrence of expanded Th17 responses (Fig. 6f). We also observed that the levels of IL-22, which can be produced by Th17 cells [42], showed no significant differences between the groups, either in spleen lymphocytes or siLPL (Additional file 2: Figure S8c). Faecal LCA was observed to be positively correlated to Th17/Treg ratio of siLPLs, and serum 6,7-diketoLCA also showed a positive correlation with the proportion of RORγt^+^T cells in the spleen (Additional file 2: Figure S9). Impaired immunological responses in the intestine are known to be associated with gut barrier dysfunction. We observed that the levels of ileal tight junction proteins such as claudin-1 and ZO-1 in the CAD group were markedly lower than those detected in the Con group (Fig. 6g). These results showed that the mice in the CAD group had weakened intestinal barrier function and intestinal permeability. Collectively, these data indicate that the faecal microbiota from CAD patients not only induced a strong systematic pro-inflammatory response but also promoted intestinal immunological activation that featured a Th17 response.

## Discussion

In this study, we demonstrated that colonization of GF mice with coronary artery disease microbiota induced profound changes in vascular stiffness. We showed that microbiota from CAD patients increased the diversity of the bile acids pool in both faeces and serum through the ability to generate ‘secondary’ bile acids. This process inhibited hepatic bile acid synthesis and caused elevated serum cholesterol levels under a high fat diet. Furthermore, colonization with CAD microbiota modified the transcriptomic profiles of genes involved in both the anti-bacteria response and mucosal inflammation pathways. The CAD microbiota increased circulatory LPS levels and pro-inflammatory cytokine expression, while it activated intestinal and systemic Th17 responses and decreased Treg cell distribution. In summary, we identified a causative role of the gut microbiota in modulating cholesterol metabolism and cardiovascular dysfunction.

In the present study, we observed that the colonization of mice with microbiota from CAD patients caused a decreased abundance of conjugated bile acids, primarily TCA, TαMAC and TβMCA, and an increased abundance of secondary bile acids, including LCA, HDCA, TDCA and keto-LCA in the faeces and serum of recipient mice. The diverse secondary BAs pool emphasized the role of the microbiota from CAD patients in modifying bile acids metabolism. Bile acids have emerged as important endocrine signalling molecules affecting lipid metabolism that can also regulate their own synthesis via nuclear receptors such as Fxr. Fgf15 is reported to be downstream signal of ileal Fxr after it is transported to the liver, where it binds Fgfr4 and is responsible for hepatic Cyp7a1 suppression. The most potent ligand for Fxr is CDCA, followed by CA, DCA, and LCA [30]. The elevated expression of Fxr and Fgf15 in Con mice may be a result of significant increases in CDCA and CA levels. We speculated that the reason no upregulation of the enterohepatic Fgf15-Fgfr4 axis observed in our study was that Fgft4 functions as insulin receptor and could be modulated by other factors [43]. Furthermore, CAD microbiota may be heavily influenced by the confounding effect of drugs, as Fgfr4 is supposed to be potential drug mediator [44]. Overall, significant increases in serum LDL-C and TC levels were observed in the CAD mice, which can be explained by a decrease in the conversion of cholesterol to bile acids in the liver, as supported by the detected downregulation of Cyp7a1 and Cyp7b1. A similar FMT study also showed that human microbiota harbour induces a phenotype of high plasma cholesterol levels that is associated with low hepatic cholesterol synthesis [45]. Collectively, these results revealed that microbiota from individuals with CAD could regulate cholesterol homeostasis via alterations in bile acids metabolism.

Metagenomic analyses of the faeces of recipient mice revealed that the microbial community and taxonomic proportions of the gut microbiota from CAD patients was transmissible. Functional analysis revealed that the CAD microbiota was less fermentative and induced more inflammation, in agreement with the results of earlier studies [35, 46]. Although active reuptake of BAs from the small intestine via the ASBT was not observed, our data suggested that Con bacteria had a stronger deconjugation ability. Importantly, in the CAD mice, we observed an increased abundance of *Clostridium symbiosum* and *Eggerthella*, enhanced 7α-dehydroxylation activity, and a strong positive correlation with faecal LCA levels. Previous studies have reported that *Clostridiaceae* and *Eggerthella* spp. have secondary BAs biotransformation activity [47]. LCA was enriched in the enterohepatic circulation of both the CAD patients and corresponding mice and may act as signalling molecule in the host. LCA is reported to be the most potent ligand for TGR5, which is another bile acid-responsive receptor involved in host metabolism [48]. TGR5 is widely recognized as a signal involved in controlling glucose homeostasis and induces NO production in vascular endothelial cells [49, 50]. In addition, the microbial HSDH-mediated generation of oxo or keto bile acids is another potential regulator of gut microbial composition and host metabolism [5]. We observed that the abundance of *Bacteroides intestinalis*, *Butyricimonas virosa* and *Pseudoflavonifractor capillosus* were significantly elevated in the CAD mice. These species harbor relatively high abundance of HDSH genes and appear to be the primary bacteria responsible for the elevated levels of 7-keto LCA in the faeces. A previous study reported that *Bacteroides intestinalis AM*-*1* isolated from human faeces is able to convert CDCA into 7-oxo-DCA and 7-oxo-LCA [51]. *Butyricimonas virosa* was also shown to be associated with cholesterol levels in the present study, although *Butyricimonas virosa* was indented as a butyric acid-producing bacterium [52]. We speculate that this discrepancy occurred because even different strains of the same bacterial species appear to have large differences in metabolic function [53]. Overall, we demonstrated that bacteria from CAD patients possessed a stronger ability to generate secondary bile acids.

The liver functions as a secondary “firewall” that protects the body from antigens crossing the intestinal barrier [54]. We observed that colonization of mice with the CAD microbiota primarily disturbed the liver immune activity with respect to cytokine production and the INF-γ response. The distribution of CD4^+^IFN-γ^+^ T lymphocytes located in the spleen in CAD mice showed an enhanced immune status as well. IFN-γ plays a crucial role in the innate immune responses that are required to limit bacterial growth and control infection [55]. In addition, IFN-γ is the most important trigger for the formation and release of reactive oxygen species (ROS), and it leads to oxidative stress development [56]. Moreover, the CAD mice showed systemic increases in pro-inflammatory factor and LPS levels, which could aggravate endotoxaemia-related chronic conditions. Taken together, the results of our study illustrated that transplantation with CAD microbiota could upregulate a systemic IFN-γ response and induce pro-inflammatory factor production.

With respect to the intestine, transcriptome analysis results provided unequivocal evidence for altered host-microbe interactions and the activation of the mucosal immune response in CAD mice that was triggered by bacterial endotoxins. The lamina propria functions as an effector in the mucosal immune system, since CD4 + T cells secrete cytokines associated with the development of intestinal inflammation [24, 57]. In our study, we observed a clear Th17 induction in the small intestine of the CAD mice, and this induction was associated with a T-cell dependent production of IL-17 and the emergence of CD4 + RORγt + T cells. Although studies have shown that *Enterococcus faecalis* colonization is capable of inducing Th17 in the intestines of mice [58]. Single species of human commensal bacteria colonization experiment, which can aid in understanding microbe-host immune relationships more comprehensively, still needs to be performed in the future. Even though we could not analyze the effect of LCA on vascular inflammation and dysfunction, a recent study reported strong evidence that derivatives of LCA can modulate the differentiation of Th17 cells, fully revealing the mechanisms through which bile acid modulates host immune responses [8]. Furthermore, the proportion of Treg cells was markedly decreased in the CAD mice and contributed to an enhanced Th17/Treg response. An abnormal ratio of Th17 and Treg cells has been frequently shown to be a key feature of chronic metabolic disease or immunological-associated disorders [59]. Collectively, our findings highlight the role of the CAD microbiota in promoting intestinal inflammation by having significant impact on Tregs and Th17 cells.

Inflammation and hypercholesterolemia are the two key aetiological factors for cardiometabolic disease. It has been demonstrated that depletion of the gut microbiota attenuates cardiac damage in experimental myocardial infarction mice [60], and the gut microbiota facilitates vascular dysfunction by supporting IL-17-mediated vascular immune cell infiltration [7]. A study of a large European cohort showed that the composition of the gut microbiota is strongly correlated with arterial stiffness in women indecent of obesity-related traits [61]. Our data showed that the CAD microbiota harbour induced higher PWV levels and collagen deposition, even compared to age-matched controls, through the FMT procedure. Moreover, CAD mice show augmented ROS levels, which can also contribute to an increased development of aortic wall stiffness. Nitric oxide (NO) is the most important endothelium-derived vasodilator molecule capable of promoting vascular health. NO breakdown by reactive oxygen species (ROS) is the primary cause of reduced NO availability and endothelial dysfunction, both in physiological ageing and arterial dysfunction [62]. Therefore, the results of the present study support a decisive role of the gut microbiota and bacterial metabolites in the regulation of vascular tone and the promotion of vascular oxidative stress.

Murine models are fundamental for investigating complex responses to the intestinal microbiota, but it is important to consider the differences in physiology and pathological between mice and humans. We used a germ-free model and increased the frequency of oral administration to ensure the long-term effect of the microbiota. Although the majority of human commensal bacteria can be established in mice, their relative compositions are altered, and a study also proved that the human microbiota may require a longer time to adjust to the environment of the mouse gut [63]. Besides, interaction between host and microbiota in sex-specific regulation of the metabolic system [64], the role of the intestinal microbiota in male models and dyslipidemic experimental models (such as ApoE^−/−^ mice), need further investigation. Regarding the limitation that bile acids are conjugated with the amino acid glycine in humans but are almost exclusively conjugated with taurine in mice, we are more concerned with respect to increasing bile salt synthesis, which leads to a relative deprivation of hepatic cholesterol content. The integrative gut microbiota-bile acid pathway has become a major target for translational and interventional studies of cardiovascular diseases. It also raises the possibility that microbiota directed therapies, including pre- or probiotic treatment, may be beneficial in treating not only atherosclerosis but also metabolic syndromes. Our findings may serve as a basis for developing markers to identify those patients who might benefit from emerging microbiota-directed therapies. Finally, this study raises an important health issue related to faecal transplantation, an increasingly popular treatment for multiple disorders.

## Conclusions

In this study, we unveil the important role of microbiota on vascular dysfunction and metabolic disorder phenotypes through faecal microbiota transplantation from CAD patients to germ-free mice. Our results reported that dysbiotic microbiota from donors could induce higher cholesterol levels by modulating secondary bile acids metabolism, especially caused the increase of LCA, TDCA, HDCA synthesis in the recipient mice. We observed that transplanted microbiota stimulated systematic endotoxemia, enhanced IFN-γ response and intestinal immune activation represented by a Th17/Treg imbalance in the mice. These findings may throw new light on the prevention of cardiovascular disease through modulating gut microbiota composition.

## Supplementary information

**Additional file 1.** Table S1 The bile acids profile in the faeces; Table S2 The bile acids profile in the serum; Table S3 Statistics for the metagenomic shotgun sequencing data of mice; Table S4 The assembly statistics of metagenomics data; Table S5 The significantly altered metagenomic pathways between Con and CAD; Table S6 Species abundance with significant differences between Con and CAD; Table S7 Differential expression of genes (DEGs) between the Con (n = 3) and CAD (n = 3) group in the liver; Table S8 Differential expression of genes (DEGs) between the Con (n=3) and CAD (n = 3) group in the ileum; Table S9 Inflammatory factors levels associated with cardiovascular disease between the GF, Con and CAD groups in the serum; Table S10 Primers for RT-PCR.

**Additional file 2.** Figure S1 Hepatic TC, TG, LDL-C and HDL-C expression levels; Figure S2 PWV measurement details; Figure S3 Microbial regulation of intestinal and hepatic genes involved in bile acid metabolism; Figure S4 Gut microbiota taxonomic and functional composition between Con and CAD mice; Figure S5 Transcriptional results of KEGG enrichment analysis of DEGs in the liver and ileum between CAD and Con groups, respectively; Figure S6 Gating strategy for the flow cytometric analyses of lymphocytes derived cells from the spleen and small intestine lamina propria. Schematic of the screening procedure; Figure S7 The cell population of total CD4^+^ T-cell and CD8^+^ T-cell in spleen and small intestine lamina propria (siLP) in Con and CAD groups n = 10–12 for each group; Figure S8 The cell population of lymphocytes in spleen and small intestine lamina propria in Con and CAD groups; Figure S9 Spearman correlation between spleen lymphocytes distributions with serum bile acids (left) and correlation between cell population of lymphocytes in siLP and fecal bile acids (right), respectively.

## Data Availability

The raw sequence data reported in this paper have been deposited in the Genome Sequence Archive (Genomics, Proteomics & Bioinformatics 2017) in National Genomics Data Center (Nucleic Acids Res 2020), Beijing Institute of Genomics (China National Center for Bioinformation), Chinese Academy of Sciences, under accession number CRA002651 that are publicly accessible at https://bigd.big.ac.cn/gsa.

## Abbreviations

CVD: Cardiovascular disease
CAD: Coronary artery disease
Con: Healthy control donor
FMT: Faecal microbiota transplantation
GF: Germ free
BAs: Bile acids
TDCA: Taurodeoxycholic acid
FXR: Farnesoid X receptor
Fgf15: Fibroblast growth factor 15
Fgfr4: Fibroblast growth factor receptor 4
siLPL: Small intestinal lamina propria lymphocytes
Treg: Regulatory T cell
ROS: Reactive oxygen species
HDCA: Hyodeoxycholic acid
UDCA: Ursodeoxycholic acid
LCA: Lithocholic acid
LPS: Lipopolysaccharide
LDL-C: Low-density lipoprotein-cholesterol
HDL-C: High-density lipoprotein cholesterol
CA: Cholic acid
CDCA: Chenodeoxycholic acid
PWV: Pulse wave velocity
