# Supplementary material for: Gut microbiota from coronary artery disease patients contributes to vascular dysfunction in mice by regulating bile acid metabolism and immune activation
Source: J Transl Med. 2020 Oct 9;18:382. doi: 10.1186/s12967-020-02539-x (PMC7547479; doi:10.1186/s12967-020-02539-x)
Supplement: Supplementary file 2 — Additional file 2. Figure S1 Hepatic TC, TG, LDL-C and HDL-C expression levels; Figure S2 PWV measurement details; Figure S3 Microbial regulation of intestinal and hepatic genes involved in bile acid metabolism; Figure S4 Gut microbiota taxonomic and functional composition between Con and CAD mice; Figure S5 Transcriptional results of KEGG enrichment analysis of DEGs in the liver and ileum between CAD and Con groups, respectively; Figure S6 Gating strategy for the flow cytometric analyses of lymphocytes derived cells from the spleen and small intestine lamina propria. Schematic of the screening procedure; Figure S7 The cell population of total CD4+ T-cell and CD8+ T-cell in spleen and small intestine lamina propria (siLP) in Con and CAD groups n = 10–12 for each group; Figure S8 The cell population of lymphocytes in spleen and small intestine lamina propria in Con and CAD groups; Figure S9 Spearman correlation between spleen lymphocytes distributions with serum bile acids (left) and correlation between cell population of lymphocytes in siLP and fecal bile acids (right), respectively. [file 12967_2020_2539_MOESM2_ESM.pdf]

Additional file 2

Gut microbiota from coronary artery disease patients contributes to vascular dysfunction in mice by regulating bile acid metabolism and immune activation

Honghong Liu[1#], Ran Tian[1#], Hui Wang[1], Siqin Feng[1], Hanyu Li[1], Ying Xiao[1], Xiaodong Luan[1], Zhiyu Zhang[1], Na Shi[2], Haitao Niu[3*], Shuyang Zhang[1*]

[1] Department of Cardiology, Peking Union Medical College Hospital, Peking Union Medical College & Chinese Academy of Medical Sciences, Beijing, China

[2] Institute of Laboratory Animal Sciences, Chinese Academy of Medical Sciences and Comparative Medicine Center, Peking Union Medical Collage, Beijing 100021, China

[3] School of Medicine, Jinan University, Guangzhou, 510632, China

[#] These authors contributed equally to this work.

*Corresponding author: Prof. Shuyang Zhang (shuyangzhang103@nrdrs.org), Department of Cardiology, Peking Union Medical College Hospital, 1 Shuaifuyuan, Dongcheng District, Beijing 100730, China. Phone: +8610-69156802

Prof. Haitao Niu (htniu@jnu.edu.cn), School of Medicine, Jinan University, Guangzhou, 510632, China.

Supplementary Figures

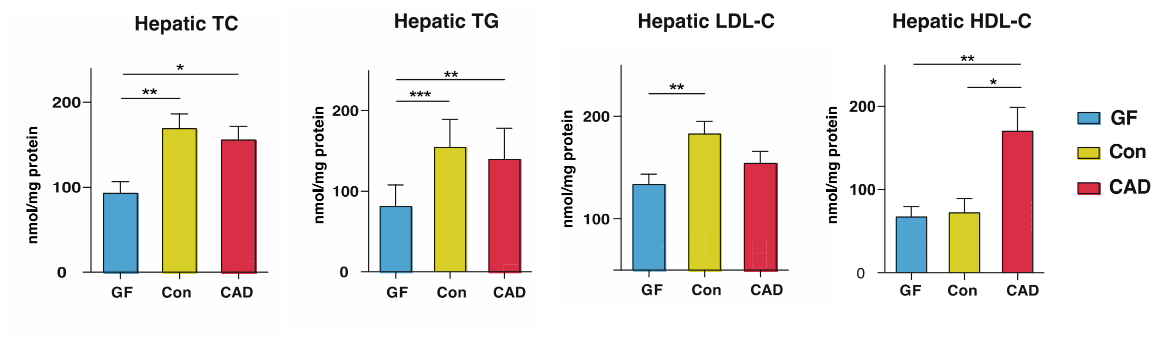

Figure S1 | Hepatic TC, TG, LDL-C and HDL-C expression levels.
Data are potted as the Means ± SEM and were compared between groups. * P < 0.05, ** P < 0.01, *** P < 0.001, Mann–Whitney U test. n = 11 or 12 for each group. TC, total cholesterol; TG, triglyceride; LDL-C, low density lipoprotein-cholesterol; HDL-C, high density lipoprotein-cholesterol. GF, germ-free mice; Con, GF mice colonized with microbiota from healthy donors; CAD, GF mice colonized with microbiota from CAD patients.

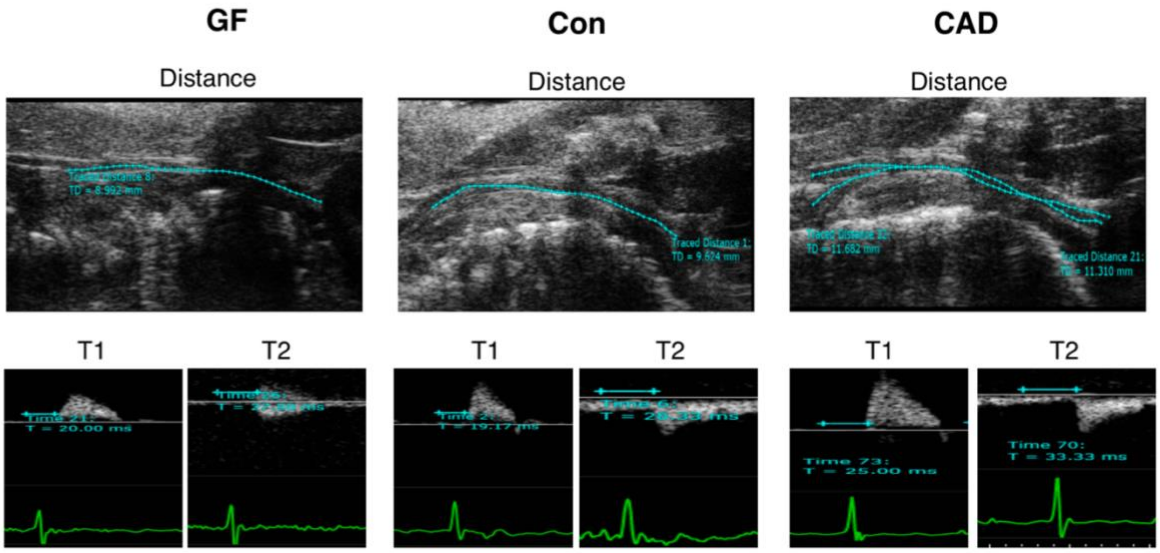

Figure S2 | PWV measurement details.
The left common carotid artery in the mouse imaged at 40 MHz, from the aortic arch to the bifurcation. Transit-time measurements were performed at distal and proximal sites, located 1.5 mm upstream from the bifurcation and 1 mm downstream from the aortic arch, respectively. Typical pulsed-wave doppler waveform measured at the proximal measurement location. The arrival time of the velocity upstroke relative to the ECG R-wave peak is denoted T1. Pulsed-wave doppler waveform observed at the distal measurement location. The arrival time is denoted T2.

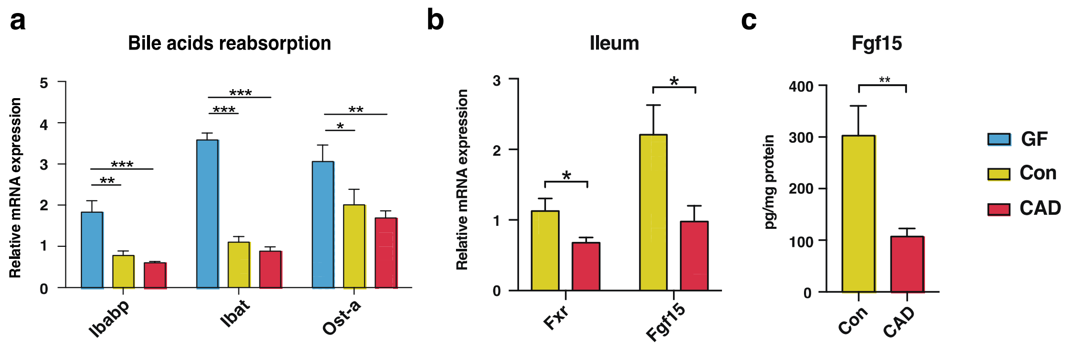

Figure S3 | Microbial regulation of intestinal and hepatic genes involved in bile acid metabolism.

a Expression of genes involved in bile acid transporters in the ileum. **b** Expression of genes involved in Fxr signaling in the ileum. **c** Ileal FGF15 protein levels. Means ± SEM are plotted; * P<0.05, ** P<0.01, *** P<0.001, Mann-Whitney U test. n = 11 or 12 for each group. GF, germ-free mice; Con, GF mice colonized with microbiota from healthy donors; CAD, GF mice colonized with microbiota from CAD patients.

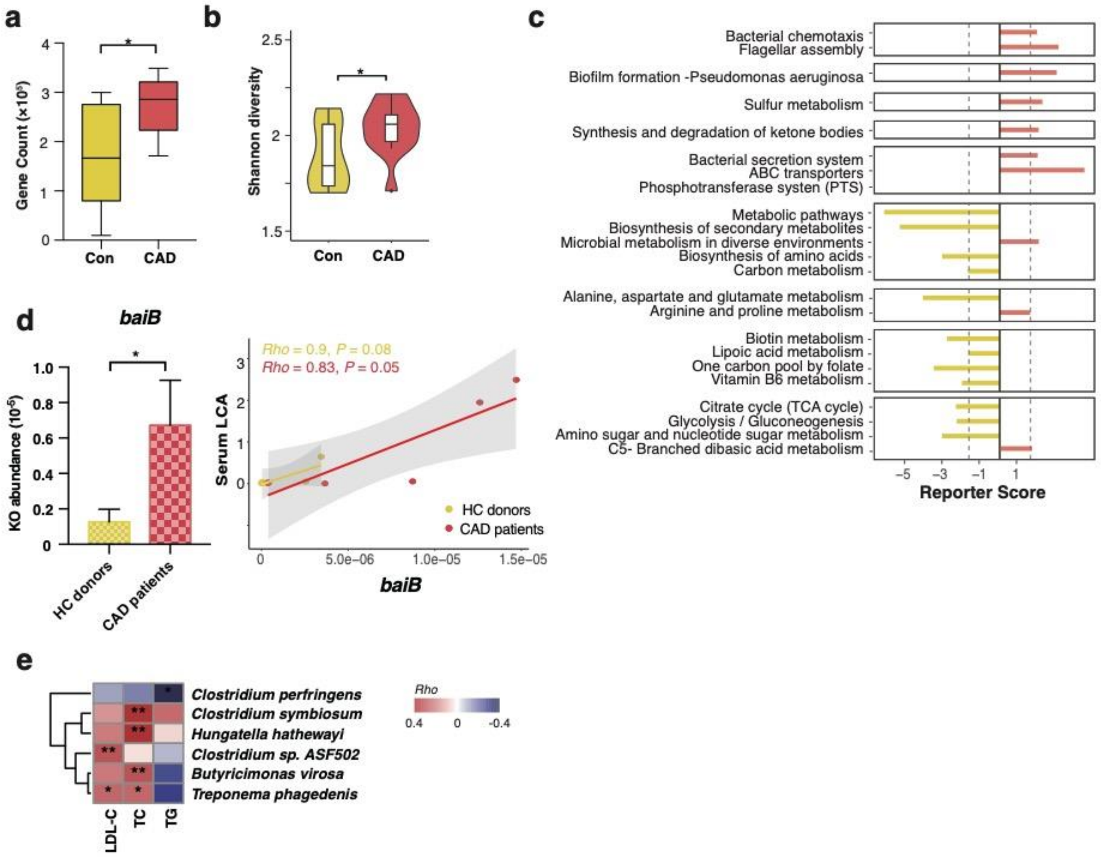

Figure S4 | Gut microbiota taxonomic and functional composition between Con and CAD mice.

a Gene richness in Con and CAD mice, boxes show the medians and the interquartile ranges (IQRs). **b** α-diversity analysis showing Con mice was characterized by lower microbial richness in Simpson indexes based on genera profiles relative. * P<0.05,

Wilcoxon rank sum test. **c** Alterations in gut microbial functional modules in Con and CAD groups. Dashed lines indicate a reporter score of 1.96, corresponding to 95% confidence in a normal distribution. **d** Boxplot of bacterial *baiB* gene abundance in HC donors and CAD patients and the spearman correlations between bacterial *baiB* gene abundance and serum LCA levels. * *P*<0.05, Wilcoxon rank sum test. **e** Spearman correlations between species abundance and serum cholesterol level. The colour represents positive (red) or negative (blue) correlations, and FDRs are denoted as follows: * FDR < 0.05, ** FDR < 0.01. n = 11 or 12 for each group.

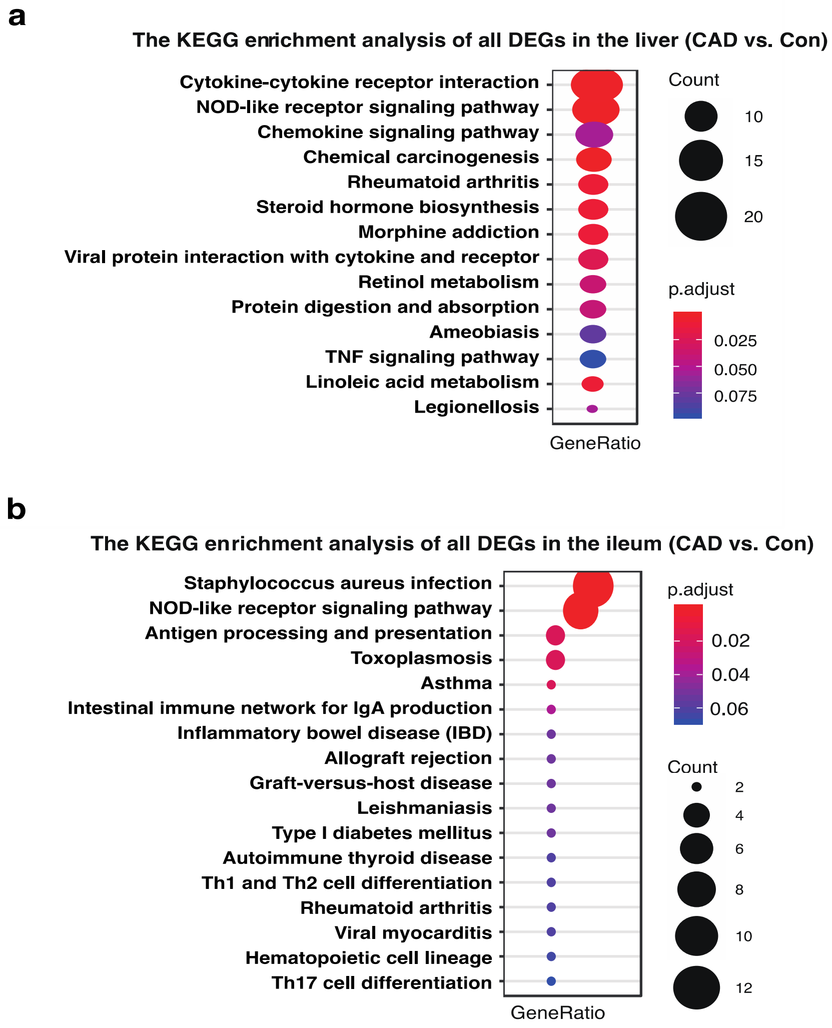

Figure S5 | Transcriptional results of KEGG enrichment analysis of DEGs in the liver and ileum between CAD and Con groups, respectively.
Dot size indicate gene ratio for each KEGG pathway. n = 3 for each group.

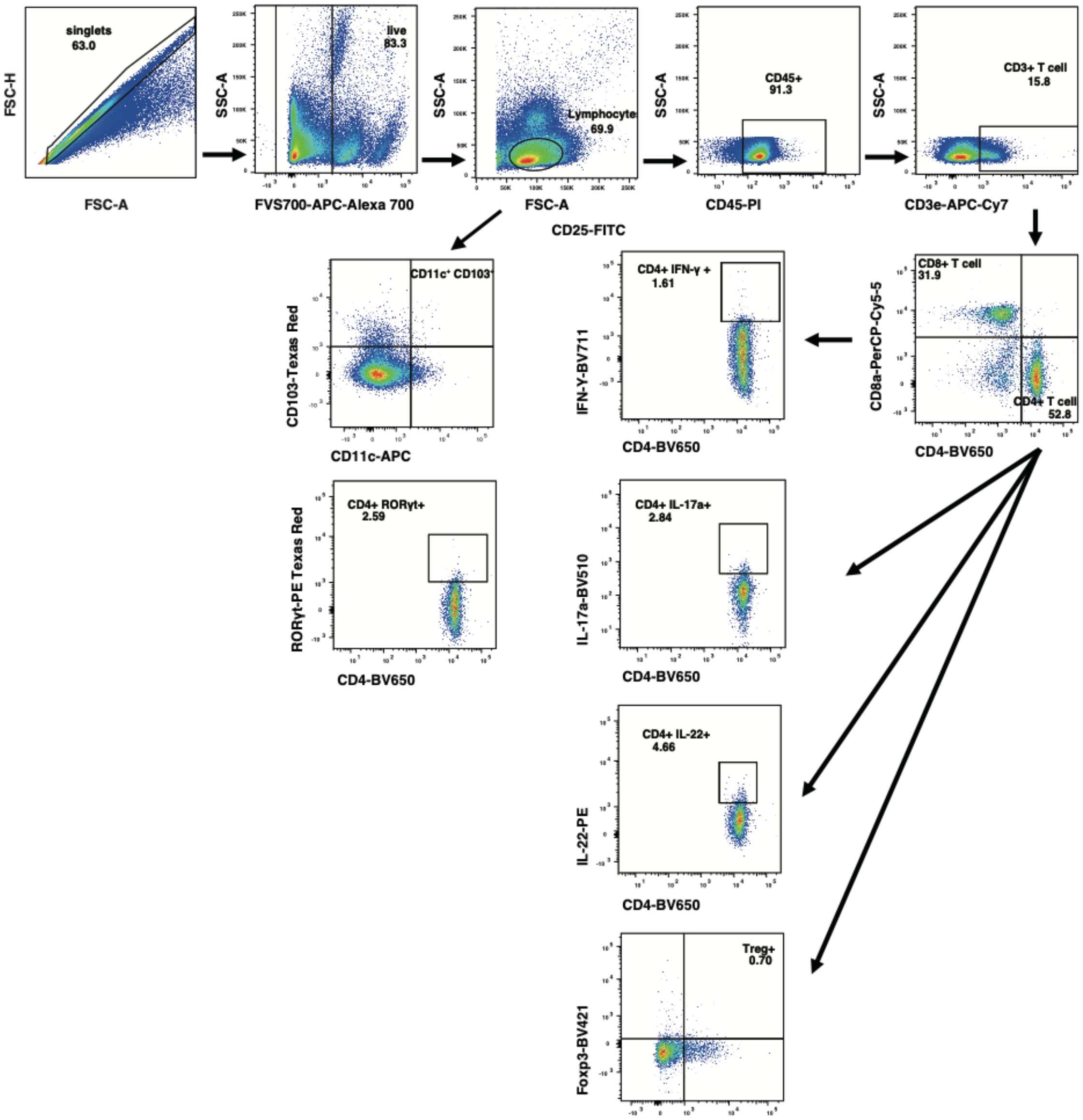

Figure S6 | Gating strategy for the flow cytometric analyses of lymphocytes derived cells from the spleen and small intestine lamina propria. Schematic of the screening procedure.

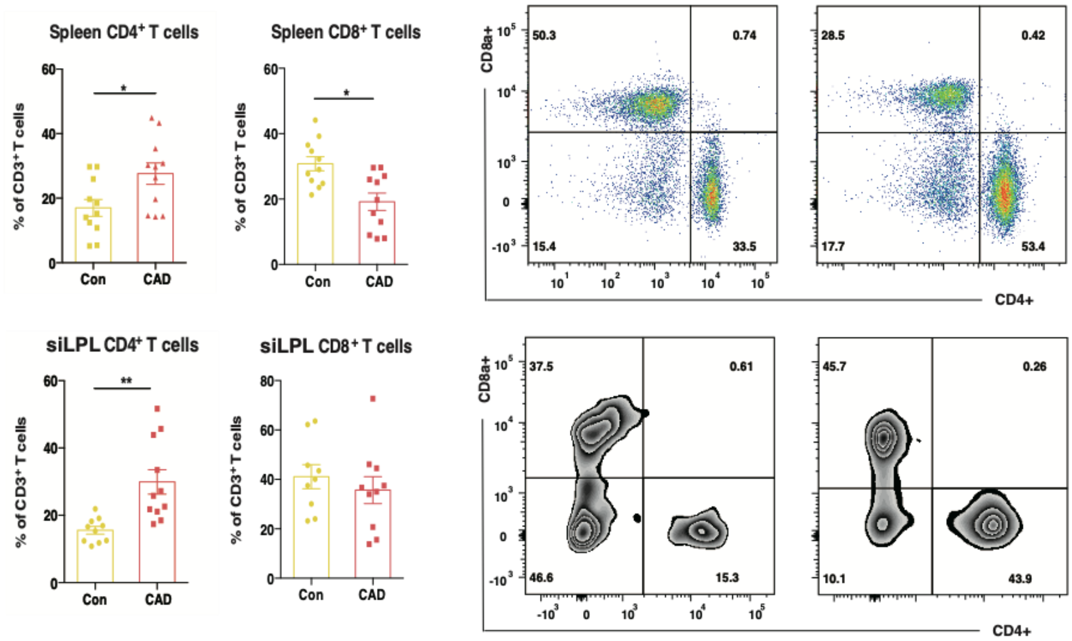

Figure S7 | The cell population of total CD4 $^+$ T-cell and CD8$^+$ T-cell in spleen and small intestine lamina propria (siLP) in Con and CAD groups. n= 10-12 for each group.

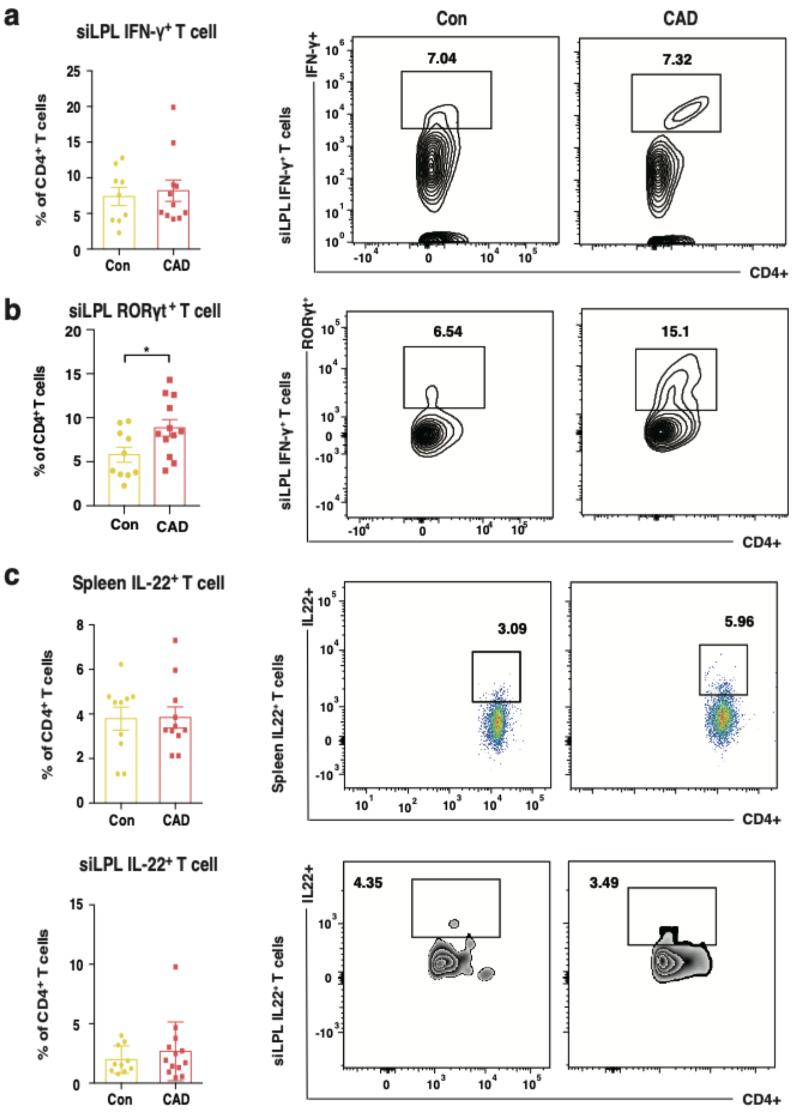

Figure S8 | The cell population of lymphocytes in spleen and small intestine lamina propria in Con and CAD groups.

a $CD4^{+}IFN\text{-}\gamma^{+}$ T cells ($CD3e^{+}CD4^{+}$ $CD8a^{-}$ $IFN\text{-}\gamma^{+}$) distributions of Con and CAD groups in small intestine. **b** $CD4^{+}ROR\gamma t^{+}$ T cells distributions of Con and CAD groups in small intestine**. c** $CD4^{+}IL\text{-}22^{+}$ T cells ($CD3e^{+}CD4^{+}$ $CD8a^{-}$ $IL\text{-}22^{+}$) proportions of Con and CAD groups in the spleen and lamina propria, respectively. n = 10-12 for each group. siLPLs, small intestine lamina propria lymphocytes.

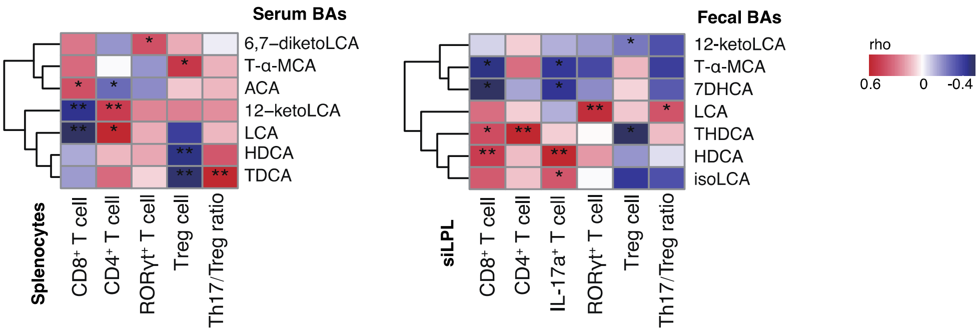

Figure S9 | Spearman correlation between spleen lymphocytes distributions with serum bile acids (left) and correlation between cell population of lymphocytes in siLP and fecal bile acids (right), respectively.
n = 10-12 for each group. The colour represents positive (red) or negative (blue) correlations, and FDRs are denoted as follows: * FDR < 0.05, ** FDR < 0.01.

Supplemental methods

Sample preparation of bile acid profiles

The stock solution of bile acids was mixed and prepared in bile acid-free serum matrix to obtain a series of bile acid calibrators at a concentration of 2500, 500, 250, 50, 10, 2.5, or 1 nM. Quality control samples were prepared in BAFUM at three different concentrations of 1500, 150, and 5 nM, respectively. Internal Standard (IS) concentrations were kept constant at all the calibration points (150 nM for GCA-d4, TCA-d4, TCDCA-d9, UDCA-d4, CA-d4, GCDCA-d4, GDCA-d4, DCA-d4, LCA-d4, and β-CA-d5). Internal standards were added to monitor the data quality and compensate for matrix effects.

UPLC-MS/MS instrument settings

UPLC	
Column Temp. (°C)	30
Sample Manager Temp. (°C)	10
Mobile Phases	A=water with formic acid (pH =3.25); and B=acetonitrile / methanol (80:20)
Gradient Conditions	0-1 min (5% B), 1-3 min (5-30% B), 3-15 min (30-100% B), 15-16 min (100-5%B), 16-17 min (5%B)
Flow Rate (mL/min)	0.40
Injection Vol. (µL)	5.0
**MASS SPECTROMETER**	
Capillary (Kv)	2.0 (ESI-)
Source Temp (°C)	150
Desolvation Temp (°C)	550
Desolvation Gas Flow (L/Hr)	1000

Analytical Quality Control Procedures

The rapid turnover of many intracellular metabolites makes immediate metabolism quenching necessary. The extraction solvents are stored in -20°C freezer overnight and added to the samples immediately after the samples were thawed. We use ice-salt bath to keep the samples at a low temperature and minimize sample degradation during sample preparation. All the prepared samples should be analyzed within 48 hours.

Reproducible and accurate results are critical for quantitative metabolomics work. To achieve this, three types of quality control samples i.e., test mixtures, stable isotope-labelled internal standards, and quality controls at three different levels (low, middle, and high) are routinely used in our metabolomics platform. In addition to the quality controls, solvent blank samples are also required for obtaining optimal instrument performance.

The BAP kit assay includes a test mixture comprising all of the bile acid reference standards. The test samples were analyzed at the beginning and end of each batch run to ensure that the instruments were performing within laboratory specifications

(retention time stability, chromatographic peak shape, and peak signal intensity). The retention time shift for a batch of 84 samples should be within 4 sec. and the difference of peak intensity should be within 15%.

Internal standards were added to the test samples in order to monitor analytical variations during the entire sample preparation and analysis processes. Three levels of QC samples (high, middle, and low concentrations) that are prepared in the BAFM are used to ensure control of each 96-well plate for BAP Ultra assay and analyzed in triplicates across the sample set. At least 67% (6 out of 9) of QC samples should be within 15% of their respective nominal value, 33% of the QC samples (not all replicates at the same concentration) may be outside 15% of nominal value but within 30%.

Reagent blank samples are a mixture of solvents used for sample preparation and are commonly processed using the same procedures as the samples to be analyzed. The reagent blanks serve as a useful alert to systematic contamination. As the reagent blanks consist of high purity solvents and are analyzed using the same methods as the study samples, they are also used to wash the column and remove cumulative matrix effects throughout the study.

The calibrators consist of a blank sample (matrix sample processed without internal standard), a zero sample (matrix sample processed with internal standard), and a series of seven concentrations covering the expected range for the metabolites present in the specific biological samples. LLOQ and ULOQ are the lowest and highest concentration of the standard curve that can be measured with acceptable accuracy and precision.
